# Organic Trace Elements Improve the Eggshell Quality via Eggshell Formation Regulation during the Late Phase of the Laying Cycle

**DOI:** 10.3390/ani14111637

**Published:** 2024-05-30

**Authors:** Songfeng Yang, Haibin Deng, Jiahao Zhu, Yiru Shi, Junyi Luo, Ting Chen, Jiajie Sun, Yongliang Zhang, Qianyun Xi

**Affiliations:** 1 Guangdong Provincial Key Laboratory of Animal Nutrition Control, National Engineering Research Center for Breeding Swine Industry, College of Animal Science, South China Agricultural University, No. 483 Wushan Road, Guangzhou 510642, China; 15270918532@163.com (S.Y.); denghaibin@stu.scau.edu.cn (H.D.); zjh0926@stu.scau.edu.cn (J.Z.); shiyiru00@163.com (Y.S.); luojunyi@scau.edu.cn (J.L.); allinchen@scauedu.cn (T.C.); jiajiesun@scau.edu.cn (J.S.); 2 Guangdong Xingtengke Biotechnology Co., Ltd., Zhaoqing 526000, China

**Keywords:** organic trace element, eggshell quality, eggshell ultrastructure, laying hen

## Abstract

**Simple Summary:**

Eggshell quality is an important factor affecting the egg production industry. Organic trace elements have been proven to delay egg spoilage. However, the potential mechanism of organic trace elements is still unclear. This study investigated the effects of different doses of organic trace elements supplementation in the diet on eggshell quality in the late phase of the laying hens. The results showed that 30–40% organic trace elements supplementation improved the eggshell quality via regulating enzymes and genes of eggshell formation.

**Abstract:**

The quality of eggshells is critical to the egg production industry. The addition of trace elements has been shown to be involved in eggshell formation. Organic trace elements have been found to have higher biological availability than inorganic trace elements. However, the effects of organic trace elements additive doses on eggshell quality during the laying period of commercial laying hens required further investigation. This experiment aims to explore the potential mechanisms of different doses of organic trace elements replacing inorganic elements to remodel the eggshell quality of egg-laying hens during the laying period. A total of 360 healthy hens (Lohmann Pink, 45-week-old) were randomly divided into four treatments, with six replications per treatment and 15 birds per replication. The dietary treatments included a basal diet supplemented with inorganic iron, copper, zinc and manganese at commercial levels (CON), a basal diet supplemented with organic iron, copper, zinc and manganese at 20% commercial levels (LOT), a basal diet supplemented with organic iron, copper, zinc and manganese at 30% commercial levels (MOT), and a basal diet supplemented with organic iron, copper, zinc and manganese at 40% commercial levels (HOT). The trial lasted for 8 weeks. The results of the experiment showed that the replacement of organic trace elements did not significantly affect the production performance of laying hens (*p* > 0.05). Compared with inorganic trace elements, the MOT and HOT groups improved the structure of the eggshells, enhanced the hardness and thickness of the eggshells, increased the Haugh unit of the eggs, reduced the proportion of the mammillary layer in the eggshell, and increased the proportion of the palisade layer (*p* < 0.05). In addition, the MOT and HOT groups also increased the enzyme activity related to carbonate transport in the blood, the expression of uterine shell gland-related genes (CA2, OC116, and OCX32), and the calcium and phosphorus content in the eggshells (*p* < 0.05). We also found that the MOT group effectively reduced element discharge in the feces and enhanced the transportation of iron (*p* < 0.05). In conclusion, dietary supplementation with 30–40% organic micronutrients were able to improve eggshell quality in aged laying hens by modulating the activity of serum carbonate transport-related enzymes and the expression of eggshell deposition-related genes.

## 1. Introduction

With the development of genetic and nutritional technologies for laying hens, the lifespan of laying hens has improved, allowing more eggs to be produced in a single production cycle [1]. However, as egg weights gradually increase with longer laying cycles, the carbonate deposition capacity of the oviductal shell gland ducts decreases, and eggshell thickness and strength decrease, thereby increasing the risk of eggshell rupture [2,3]. The eggshell quality is a critical feature to affect egg marketing in the commercial poultry industry [3]. It is reported that the economic losses of cracked and broken eggs account for 10% of total production [4]. In addition, eggshell defects increase the risk of eggs being exposed to the environment, resulting in shorter egg shelf life. Therefore, ways to improve eggshell quality in the later stages of laying hens are essential to increase the commercial value of laying hens.

The quality of eggshells is influenced by the linear mineralization process which is characterized by the initiation of calcium deposition at the papillae of the shell membrane, the extension of calcite crystals and ending below the cuticle [5]. Relevant studies showed that the ordered crystal structure of the palisade layer and papillae layer is crucial for maintaining the strength and thickness of the eggshell [6]. Trace elements are involved in animal growth and development, including bone formation and eggshell deposition, and are very important for laying hens [7]. Copper, manganese and zinc are essential for the formation of eggshells and eggshell membranes, possessing catalytic properties as key enzymes and interacting with calcite minerals to increase eggshell strength and thickness by improving the ultrastructure of the eggshell [8,9]. Iron plays an important role in shell pigmentation of brown eggs [10]. It is reported that the supply of mineral nutrients such as zinc and manganese in the diet alleviated the insufficient turnover of elements in the later stage of the laying cycle, maintained the stability of the eggshell submicroscopic structure, increased the effective thickness of the eggshell, and finally improved the eggshell quality [11]. A previous study showed that dietary supplementation with inorganic or protein manganese, zinc and copper increased effective thickness, and eggshell strength [12]. Mechanistically, on the one hand, traces elements as coenzyme factors participate in the expression of calcium deposition-related enzyme activities during the eggshell formation process [13]. On the other hand, traces elements participate in maintaining the specific substructures of eggshells [14]. However, traditional inorganic minerals supplied in the diet have low bioavailability and dose-dependent toxic effects, increasing the risk of environmental pollution [15]. To avoid these problems, organic trace elements have been used to replace the inorganic trace minerals applied in the diet [15]. Some previous studies have showed that amino acid-derived trace element supplement in the diet reshaped the eggshell structure [12,16]. A recent study showed that the peptide chelation form had a better effect on absorption and stability than amino acid-chelated trace element and a low dose of protein-chelated multiple trace elements improved the eggshell quality of laying hens in the late stage of egg production [17]. Considering the expensive commercial price of organic trace elements, the dose and mechanism of peptide-chelated trace elements supplemented in the diet completely replacing inorganic trace minerals still needs further investigation.

The aim of this study was to comprehensively assess the effect of peptide-chelated trace elements on egg production performance, eggshell quality, eggshell ultrastructure, and trace element deposition in aged laying hens and to explore the mechanism of peptide-chelated trace elements regulating eggshell quality.

## 2. Materials and Methods

### 2.1. Animal Ethics Statement

All experimental protocols were approved by the Animal Care Institution and Ethics Committee of South China Agriculture University. All animal experiments adhered to the animal experiment policy of South China Agriculture University, China (SYXK2014-0136).

### 2.2. Birds and Management

A total of 360 healthy hens (Lohmann Pink, 45-week-old) with similar weights (1677 ± 112 g) were randomly divided into 4 treatments, with 6 replications per treatment and 15 birds per replication. The dietary treatments included a basal diet supplemented with 30, 10, 70, and 80 mg/kg inorganic Fe, Cu, Zn, and Mn from sulphates (FeSO_4_, Cu_2_(OH)_3_Cl, ZnSO_4_, an MnSO_4_, provided by Guangdong Xingtengke Biotechnology Co., Ltd., Zhaoqing, China) according to commercial levels (CON; 100%), a basal diet supplemented with organic mineral at 20% commercial levels (LOT; 6, 2, 14, 16 mg/kg organic Fe, Cu, Zn, and Mn (provided by Guangdong Xingtengke Biotechnology Co., Ltd., Zhaoqing, China)), a basal diet supplemented with organic mineral at 30% commercial levels (MOT; 9, 3, 21, 24 mg/kg organic Fe, Cu, Zn, and Mn), and a basal diet supplemented with organic mineral at 40% commercial levels (HOT; 12, 4, 28, 32 mg/kg organic Fe, Cu, Zn, and Mn). The basal diet was formulated according to NRC (1994) [18] recommendation and Chinese standards (2004) [19] to meet the requirement of laying hens. The composition and nutrition level of diets were shown in Table 1. The experiment lasted for 8 weeks, and during the experimental period, laying hens were housed in three-tiered conventional wise bird cages (45 cm length × 70 cm width × 30 cm height), with three laying hens per cage, given free access to water and diets, and a 16 h light cycle at 24 °C management schedule was used. The feed formulation and chemical composition were shown in Table 1.

### 2.3. Sample Collection

All eggs produced by laying hens were collected at 4:00 p.m. every day over the whole trial to analyze the egg quality. The egg production number and weight in each replicate were recorded to calculate the egg production, daily egg mass, and egg loss according to a procedure published previously [12]. The feed consumption was statistically recorded every week to determine the average daily intake and feed-to-egg ratio of laying hens. The fecal samples were collected by installing plastic baffles under the cage in the last two days of the experimental period. At the end of the experiment, all laying hens were fasted from food for 12 h to collect blood samples. The blood samples were centrifuged at 3000× *g* for 15 min at 4 °C to obtain the serum. All serum samples were stored at −20 °C immediately for future analyses. Laying hens were euthanized by cervical dislocation after the blood samples collection. The shell gland of the oviduct was collected and gently flushed with PBS (phosphate buffer saline) to remove secretion. Then, the samples were divided into two parts, fixed with 4% polyformaldehyde or frozen in liquid nitrogen, respectively.

### 2.4. Blood Parameter Analysis

Serum Ca, P, uric acid (UA), glucose (GLU), total cholesterol (T-CHO), triglyceride (TG), albumin (ALB), glutamine aminotransferase (AST), alanine transaminase (ALT), total antioxidant capacity (T-AOC), total superoxide dismutase (T-SOD), Cu/Zn-SOD, Mn-SOD, glutathione peroxidase (GSH-Px), catalase (CAT), malondialdehyde (MDA), alkaline phosphatase (ALP) and ceruloplasmin were measured by commercial kits (Jiancheng Bioengineering Institute, Nanjing, China) according to the manufacturer’s instructions protocol. Serum estradiol (E2), carbonic anhydrase (CA), lysyl oxidase (LOX), transferrin, IgA, IgM, and IgG were measured using ELISA kits (Jiangsu Meibiao Biotechnology, Yancheng, China) according to the instructions provided by the company.

### 2.5. Eggs Parameter Analysis

Egg Analyzer EA-01 (Orka Food Technology, Herzliya, Israel) was used to measurement the egg weight, Haugh unit, albumen height, and yolk color. Then the egg yolk was stored at −20 °C to measure the concentration of the trace mineral. The eggshell breaking strength was measured using an Egg Force Reader (YN-100, Yaoen Instrument Equipment Co., Ltd., Nanjing, China). The eggshell thickness was measured both at the blunt end, tip, and equator using a micrometer after removing the eggshell membrane and then the average was calculated followed by previous reports [21].

### 2.6. Eggshell Ultrastructure Observation

The eggshell fragments (5 mm × 5 mm) were selected for scanning electron microscope analysis (EVO MA 15, ZEISS, Oberkochen, Germany). Eggshell samples were used for the cross section of the eggshell observation. As described previously, the eggs were broken and the eggshell from the equator was softly wash with ddH_2_O and then dried at room temperature for 48 h [22]. Then, the fragment was immobilized vertically to the aluminum support and sprayed with gold powder. For each eggshell sample, we randomly selected three different fields of view to measure the mammillary layer thickness, palisade layer thickness, and mammillary knob width. For internal surface observation, the inner membrane of eggshell was removed.

### 2.7. Histopathological Analysis

The shell gland of the oviduct was fixed in 4% paraformaldehyde for 24 h and then dehydrated with ethanol, transparentized with xylene, embedded in paraffin, and sectioned into 5 μm thick slices (Service Biotechnology, Wuhan, China). Subsequently, the hematoxylin and eosin solution were (Service Biotechnology, Wuhan, China) used to stain the slide. Stained uterine tissue sections were examined microscopically using an OLYMPUS BX43 microscope (Olympus Corporation, Beijing, China), and the height of the uterine cilia was measured in three randomly selected fields of view using Image J 1.53.

### 2.8. Trace Elements Measurement

The samples of fresh feces and eggshell about 1 g were carbonized in an adjustable electric heating plate at 200 °C until the sample turned smoke-free. The carbonized samples were ashed in a muffle furnace at 550 °C for 3–4 h. In total, 10 mL of mixed acid solution (concentrated nitric acid and perchloric acid, 9:1, *v*/*v*) was added into the sample (concentrated yolk and ashed samples) and digested for 2 h at 160 °C as previous described [23]. The trace element concentration was determined by an inductively coupled plasma optical emission spectrometer (Optima 7300 DV; PerkinElmer, Waltham, MA, USA).

### 2.9. Real-Time Quantitative PCR

According to the manufacturer’s instructions, the total RNA was separated from the shell gland of the oviduct using TRIZOL reagent (Thermo Scientific, Waltham, MA, USA). Reverse transcription of the total RNA was performed using a Color Reverse Transcription Kit (EZBioscience, Roseville, CA, USA). Real-time quantitative PCR was performed using 2 × RealStar SYBR Mixture (A301, Genstar, Beijing, China). β-actin was used as a reference for normalization. Primers listed in Table 2 were designed using Primer-Blast and the 2^−ΔΔCT^ method was performed to analyze the relative quantification. 

### 2.10. Statistical Analyses

SPSS 22.0 (IBM, Armonk, NY, USA) was used to analyze the data. Data were analyzed by one-way ANOVA. The parametric Tukey test was considered for multiple comparisons. Data were expressed as mean ± SEM. *p* < 0.05 indicated significant differences.

## 3. Results

### 3.1. Organic Trace Elements Supplement Had No Impact on Performance of Laying Hens

The egg production, average daily feed intake, daily egg mass, feed-to-egg ratio, and egg loss were shown in Table 3. During the experiment, different doses of organic trace elements supplement in experimental diets had no effect on productive performance of laying hens (*p* > 0.05). As presented in Table 4, compared with the CON group, different doses of organic trace elements supplement in the diet had no significant changes in the concentration of serum metabolites such as UA, GLU, T-CHO, and TG (*p* > 0.05). As presented in Table 5, organic trace elements supplement did not result in abnormal changes in liver function-related damage indices proteins ALB, AST, and ALT (*p* > 0.05). In Table 6, the concentrations of serum immunoglobulins (IgA, IgM, and IgG) were not significantly different among all groups (*p* > 0.05). Although there were no significant differences in serum antioxidative indicators such as T-SOD, Cu/Zn-SOD, Mn-SOD, GSH-Px, and MDA among all groups, the serum CAT level was greater after the organic trace elements supplement in the diet compared with the CON group in Table 7 (*p* < 0.05). Additionally, the T-AOC activity in the MOT group was higher than that in the LOT and HOT groups (*p* < 0.05).

### 3.2. Organic Trace Elements Supplement Delayed Quality Deterioration of Eggs

In our experiment, the effect of egg quality traits was more pronounced with increasing concentrations of organic trace elements. Briefly, we found that organic trace elements supplement had no effect on the yolk color (*p* > 0.05), while the albumen height, Haugh unit, shell strength, and eggshell thickness were greater in MOT and HOT treatments (*p* < 0.05) (Table 8).

### 3.3. Organic Trace Elements Supplement Improved Eggshell Quality of Laying Hens

**Figure 1:**
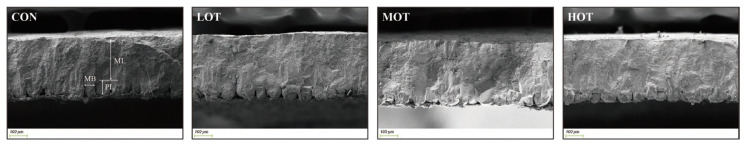
Effect of organic trace elements supplement on scanning electron microscope images of eggshell cross ultrastructure of laying hens at 300× magnifications with a 100 μm ruler. CON: 100% commercial level inorganic trace elements; LOT: 20% commercial level organic trace elements; MOT: 30% commercial level organic trace elements; and HOT: 40% commercial level organic trace elements. Abbreviations: PL, Palisade layer; ML, mammillary layer; and MB, vertical view of the mammillary buttons.

To further investigate whether organic trace elements supplement in the diet enhance eggshell strength and thickness, we observed the microstructure of the eggshell using a scanning electron microscope. As shown in Figure 1 and Table 9, compared with the CON group, the mammillary layer thickness was lower in MOT and HOT groups while the total thickness, palisade layer thickness, mammillary knob width, and the ratio of mammillary layer to palisade layer of the eggshell was greater (*p* < 0.05). However, the LOT group was not significantly different in terms of improving the ultrastructure of the eggshell than the CON group (*p* > 0.05).

### 3.4. Organic Trace Elements Supplement Promoted Eggshell Formation of Laying Hens

**Figure 2:**
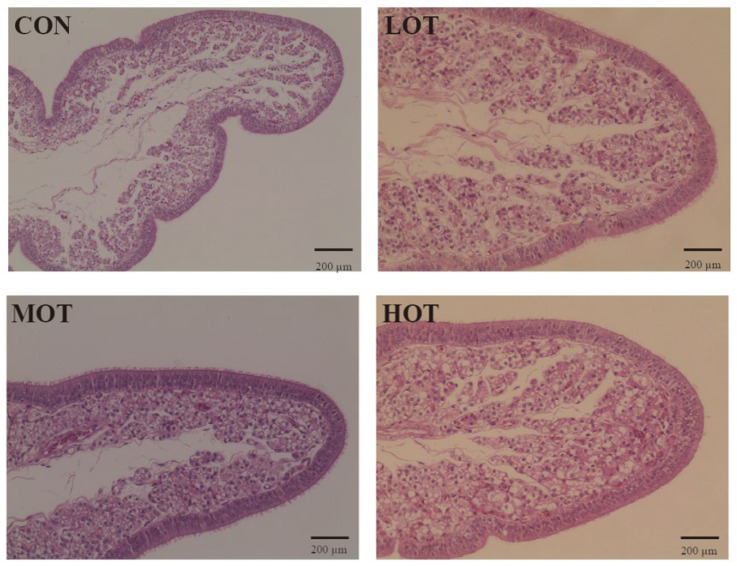
The histological structure of the uterus in laying hens at 40× magnifications with a 200 μm ruler. CON: 100% commercial level inorganic trace elements; LOT: 20% commercial level organic trace elements; MOT: 30% commercial level organic trace elements; and HOT: 40% commercial level organic trace elements.

**Figure 3:**
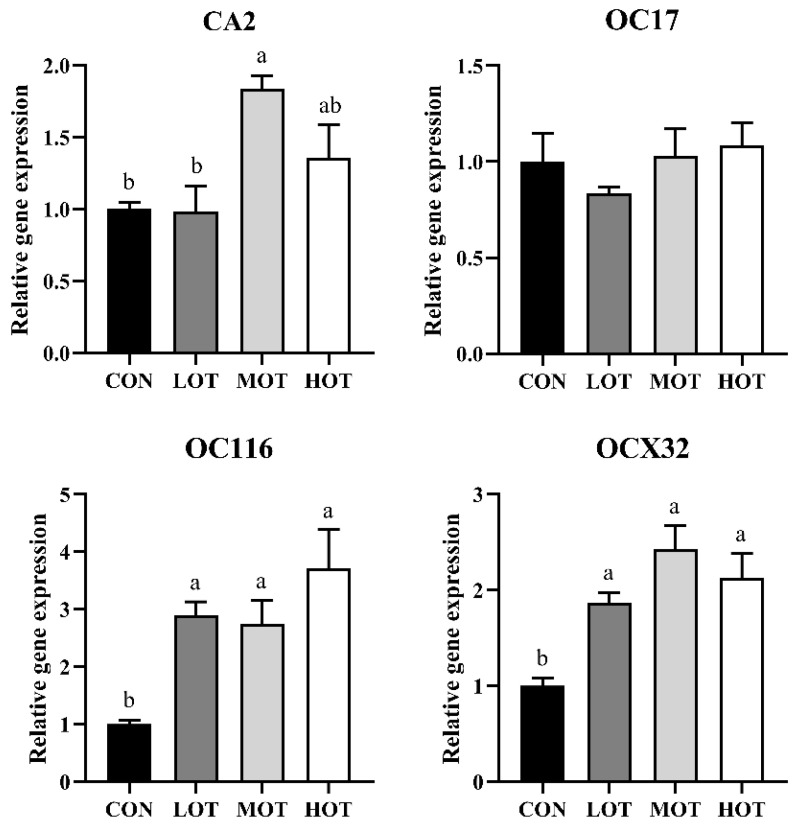
The gene expression of CA2, OC17, OC116, and OCX32 in the uterus of laying hens. Values are means ± SEM. Different markup represented significant differences (*p* < 0.05). CON: 100% commercial level inorganic trace elements; LOT: 20% commercial level organic trace elements; MOT: 30% commercial level organic trace elements; and HOT: 40% commercial level organic trace elements. CA2: carbonic anhydrase II; OC17: ovocleidin-17; OC116: ovocleidin-116; and OCX32: ovocalyxin-32.

The formation of eggshell is a complex process that involves multiple enzymes and hormone coordination. Thus, we measured the serum enzyme activities involved in eggshell formation as performed in Table 10. Compared with the CON group, the serum LOX concentration was lower in the LOT group (*p* < 0.05), while this in the HOT group was greater compared with the CON group (*p* < 0.05). The enzyme activity of CA was influenced by organic trace elements. Compared with the CON group, the MOT and HOT group showed a higher CA level in the serum (*p* < 0.05). Moreover, the HOT group had a high ALP level in the serum than the CON group (*p* < 0.05). Notably, the activities of these serum enzymes associated with calcium carbonate deposition are regulated by the level of hormones. However, the concentration of E2 in the serum did not change in the experiment (*p* > 0.05). The shell gland of the oviduct is a critical organ for eggshell formation, and the integrity of the structure is fundamental for its function. As shown in Figure 2 and Table 11, the number of shell gland duct of oviducts was increased with the organic trace elements supplement. In addition, compared with the CON group, the uterus cilia length in the MOT and HOT groups were shorter (*p* < 0.05). Next, we determined eggshell formation-related gene expression of the oviduct in Figure 3. Similar to the change of CA concentration, the MOT group showed a higher CA2 level in the serum compared with the CON and LOT groups (*p* < 0.05). Moreover, organic trace elements supplement significantly increased the gene expression of ovocleidin-116 (OC116) and ovocalyxin-32 (OCX32) in the oviduct (*p* < 0.05), while the gene expression of ovocleidin-17 (OC17) did not change in any of the groups (*p* > 0.05). The deposition of calcium and phosphorus determined the fate of the eggshell. To further confirm whether organic trace elements supplement promote eggshell formation, the content of calcium and phosphorus in the serum and eggshell were measured respectively in Table 12. Organic trace elements supplement had no effect on the concentration of calcium and phosphorus in the serum (*p* > 0.05). Compared with the CON and LOT group, the MOT and HOT groups showed a higher calcium concentration in the eggshell (*p* < 0.05), while the concentration of phosphorus did not change in any of the groups (*p* > 0.05).

### 3.5. Organic Trace Elements Supplement Reduced Trace Elements Residual in Feces and Serum

**Figure 4:**
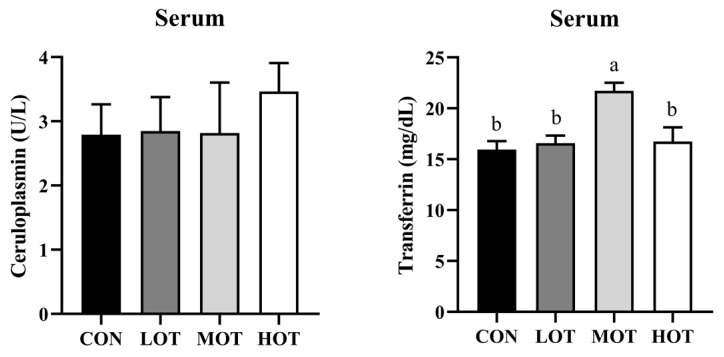
Effect of organic trace elements supplement on the activity of transferrin and ceruloplasmin in the serum of laying hens. Data were the mean of six replicates per treatment. Values are means ± SEM. Different markup represented significant differences (*p* < 0.05). CON: 100% commercial level inorganic trace elements; LOT: 20% commercial level organic trace elements; MOT: 30% commercial level organic trace elements; and HOT: 40% commercial level organic trace elements.

Next, we investigated the deposition patterns of organic trace elements in laying hens. As shown in Table 13, organic trace elements supplement in the diet significantly reduced the residual of Fe, Cu, Zn, and Mn in feces (*p* < 0.05). Considering the dose of organic trace elements supplement was increased in treatment groups, the LOT and MOT group presented lower trace elements deposition than HOT in feces (*p* < 0.05), while the LOT and MOT group had no difference (*p* > 0.05). Similar to the change of trace elements deposition patterns in feces, the LOT group had the lowest level of organic trace elements concentration in the serum (*p* < 0.05). In addition, the MOT and HOT groups did not change the Cu and Mn concentration in the serum compared with the CON group (*p* > 0.05). The concentration of iron in the serum was lower after organic trace elements were supplied in the diet (*p* < 0.05). As presented in Figure 4, the MOT group showed a higher activity of transferrin in the serum (*p* < 0.05), while the ceruloplasmin concentration did not change in any of the groups (*p* > 0.05). Unfortunately, we did not detect the effective content of Mn in the serum and eggshell, which may be related to the detection threshold. Furthermore, the MOT group showed a lower Cu concentration in the eggshell (*p* < 0.05), while the other indicators showed that organic trace elements had no effect on yolk and eggshell deposition (*p* > 0.05). This evidence indicated that, the residual of trace elements in feces was lower after the MOT supplement in the diet improved the utilization of iron.

## 4. Discussion

Organic trace elements are structurally stable and do not dissociate easily before absorption, and after entering the digestive tract, they can avoid being absorbed by precipitates in the intestinal lumen (e.g., phytic acid, phosphoric acid, oxalic acid, etc.) [25]. In addition, small peptide-chelated organic trace elements are transported and absorbed in the form of amino acids, which reduces antagonism and competition for binding sites with other inorganic trace elements, thus demonstrating advantages over inorganic trace elements [12,26]. Our study indicates that low levels of organic trace elements added to diets in place of inorganic micronutrients do not negatively impact the production performance. Specifically, Organic trace elements at 20%, 30%, and 40% commercial levels were effective in maintaining production indicators, such as egg production rate, similar to those of inorganic trace elements at 100% commercial levels, which is in line with previous research reports [8]. Serum levels of UA, GLU, T-CHO, and TG were indicators of animal health. Trace elements are associated with lipid metabolism, and deficiencies may cause disorders of lipid metabolism, which are usually manifested by elevated serum lipid metabolism-related markers [27]. In this study, low levels of organic trace elements replacement did not significantly affect these indicators. The results of Aksu’s study equally showed that lower levels of organic trace elements did not negatively affect serum biochemical indices in broilers [27]. When tissue cells are damaged or necrotic, Alanine aminotransferase (ALT) and Aspartate transaminase (AST) are released and their activity in the serum is elevated [28,29]. It has been reported that 1/3 NRC recommended levels of organic trace elements do not significantly affect serum ALT and AST activities in livestock [27], which is consistent with the results obtained in our experiment. Immunoglobulin A (IgA), Immunoglobulin M (IgM) and Immunoglobulin G (IgG), are immune response antibodies produced by bone marrow-dependent lymphocyte. Trace elements act as activators that indirectly influence the content of immune proteins [30]. There was no significant difference in the immunological indices of all treatments in this experiment. In addition, there were no significant differences in serum antioxidant indicators such as T-SOD, Cu/Zn-SOD, Mn-SOD, GSH-Px, and MDA among the groups. However, compared with the CON group, the serum CAT level was improved more significantly after organic trace elements supplement in the diet. In fact, serum CAT activity, as the only antioxidant indicator that differed in this trial, is not comprehensive enough to reflect the ability of organic trace elements’ replacement to improve antioxidant capacity in late laying hens. Trace elements are the active components of many antioxidant enzymes, and the lack of any one element will lead to the reduction of enzyme activity, thus affecting the biological body’s function of scavenging superoxide free radicals and reducing its resistance to oxidative toxicity [31,32,33]. Therefore, the results of this experiment suggest that different levels of organic trace elements replacing inorganic trace elements have no negative effects on the normal physiological and metabolic functions of laying hens.

The egg quality index is related to the quality of the eggshell [34]. When the eggshell quality is at a low level, the protective effect of the eggshell on the egg is weakened due to the decrease in the effective thickness of the eggshell, the egg becoming fragile, and the risk of harmful bacteria infection increasing [35]. In our experiment, the effect of egg quality traits was more pronounced with increasing concentrations of organic trace elements. Briefly, we found that organic trace elements supplement had no effect on the yolk color, while the MOT and HOT supplement in diet significantly increased albumen height, Haugh unit, shell strength, and eggshell thickness, which delayed the quality deterioration of eggs. These findings are in accordance with the findings reported by Stefanello [12]. Further analysis of the eggshell submicroscopic structure revealed that, compared with the CON group, the mammillary layer thickness was lower in MOT and HOT groups while total thickness, palisade layer thickness, mammillary knob width, and the ratio of mammillary layer to palisade layer of the eggshell were greater. However, the LOT group was not significantly different in terms of improving the ultrastructure of the eggshell than the CON group. Relevant studies have shown that the total thickness of the eggshell, the ratio of the fenestrae to the papillae, and the density and width of the papillae nodes affect the strength of the eggshell, with the longer the length of the fenestrae and the shorter the length of the papillae, the greater the ability of the eggshell to resist external forces, and the greater the strength of the eggshell [36,37]. Therefore, these results confirm that 30% and 40% commercial levels of organic trace elements improve eggshell quality by modulating the eggshell submicroscopic structure. However, the effect of the 20% commercial level of organic trace elements supplementation was similar to that of the CON group, and even worse than that of the CON group in terms of eggshell strength, which might be caused by the low level of organic trace elements supplementation in the LOT group. Copper, manganese and zinc are promoters or components of enzymes such as Lysyloxidase (LOX) and Carbonicanhydrase (CA), respectively, which are involved in the formation of eggshells [38,39,40]. LOX is a copper-containing enzyme involved in the conversion of lysine to a variety of proteins within the eggshell membrane and plays a key role in the formation of the eggshell membrane [38,41]. CA is a zinc-containing enzyme that catalyzes the hydrolysis of carbon dioxide to form the calcium carbonate precursor of the eggshell, which in turn leads to the deposition of calcium carbonate crystals to form eggshells [42,43]. Compared with the CON group, the serum LOX concentration was lower in the LOT group, while this in the HOT group was greater compared with the CON group. However, the MOT and HOT group showed a higher CA level in the serum compared with the CON group. Estrogen (E) is a steroid hormone produced primarily by the ovaries that exerts its biological effects by binding to receptors and influencing the metabolism [44]. Alkaline phosphatase (ALP) is a zinc-containing enzyme, activated by manganese, associated with eggshell calcification [45]. In our study, the serum ALP level was significantly higher in the HOT group than in the CON group and significantly lower in the LOT group than in the MOT and HOT groups. The concentration of E in the serum had no changes in the experiment. Then, we examined the calcium and phosphorus levels in serum and eggshells and found that the calcium levels in eggshells in the MOT and HOT groups were significantly increased, which is consistent with the enzyme activity results. It is worth noting that uterine homeostasis underlies eggshell biomineralisation [46]. Previous studies have reported that, with increasing age, the length of the uterine cilia becomes long and sparse and the number of tubular gland cells in the uterine tissue decreases [47]. Histological analysis of the uterus in the present study suggested that the MOT and HOT groups delayed the decline of uterine tissues and that the uterine cilia length of these two groups was significantly lower than that of the CON and LOT groups. To further explore how organic micronutrients affect eggshell quality, we determined the expression of genes involved in eggshell deposition in uterine tissues. Previous studies have shown that, ovocleidin-17 (OC17) [48], ovocalyxin-32 (OCX32) [49], ovocleidin-116 (OC116) [50], and CA2 are the main matrix proteins secreted from uterine fluid, which are involved in eggshell mineralization [51,52]. Similar to the change of CA concentration, the MOT group showed a higher CA2 level in the serum compared with the CON and LOT groups. Moreover, the organic trace elements supplement significantly increased the gene expression of OC116 and OCX32 in the oviduct, while the gene expression of OC17 did not change in all groups. Therefore, organic trace elements, as the substitute for inorganic micronutrients, added to the diets of laying hens in the late laying stage can improve eggshell quality by increasing the activity of relevant enzymes, maintaining the morphology of uterine tissues, and increasing the expression of relevant genes.

Deposition of trace elements in animal tissues and organs is often the main indicator for assessing the bio-efficiency of trace elements in feed [53]. In this study, we measured the contents of trace element in the serum, yolk, shell and feces of laying hens to find low levels of organic trace elements substituted for inorganic trace elements added to the diets significantly reduced the excretion of Fe, Cu, Zn and Mn in the feces. Similar to the change of trace elements deposition patterns in feces, the LOT group had the lowest level of organic trace elements concentrations in the serum. In addition, the MOT and HOT groups had no effect on the Cu and Mn concentrations the in serum compared to the CON group. Furthermore, with the exception of the MOT group which reduced the Cu concentration in the eggshell, the other indicators showed that organic trace elements had no effect on yolk and eggshell deposition. The concentration of iron in the serum was reduced after organic trace elements were supplied in the diet. Notably, iron and copper existed in the serum in both the bound and free states [20,54]. The transferrin and ceruloplasmin in the serum reflected the binding activity of iron and copper, respectively [55,56]. Our study suggests that the MOT group significantly increased the activity of transferrin in the serum, while the ceruloplasmin concentration did not change in any of the groups, which may be due to the lower serum Fe concentration in the LOT, MOT and HOT groups than that in the CON group.

## 5. Conclusions

In conclusion, replacing 100% inorganic trace elements with 20% organic trace elements did not improve the quality of eggshells, although it reduced trace elements in feces, which may be related to the low level of added trace elements, whereas replacing inorganic trace elements with 30–40% organic trace elements improved the deposition of minerals in eggshells by remodeling the ultrastructure of the shells and regulating the homeostasis of the uterus, which in turn improved the quality of the eggshells.

## Figures and Tables

**Table 1:** Ingredient and nutrient composition of the basal diet.

Items	Content (%)
Ingredients (%)	
Corn	66
Soybean meal	23
Ground limestone	8.5
DL-Methionine	0.2
Premix ^1^	2.3
Total	100
Nutrient levels ^2^	
ME (MJ/kg)	11.19
Crude protein (%)	15.56
Methionine (%)	0.44
Lysine (%)	0.77
Methionine + cysteine (%)	0.71
Calcium (%)	3.87
Total phosphorus (%)	0.47
Available phosphorus (%)	0.25
Analysed value ^3^ (mg/kg)	
Fe	89.52
Cu	18.75
Zn	30.7
Mn	10.64

^1^ The premix without trace minerals contains the following per kg of the diet: VA 11000 IU, VD3 4388 IU, VE 23 IU, VK 2.6 mg, thiamine 1.6 mg; riboflavin, 8.1 mg; folic acid, 1.3 mg; niacin acid, 39 mg; pyridoxine, 4 mg; biotin 0.065 mg; vitamin B12, 0.02 mg; calcium pantothenate 11 mg; choline chloride 375 mg. ^2^ Crude protein and calcium values were analyzed. The other nutrient levels were calculated. Amino acid levels are expressed on a total basis. ^3^ According to AOAC Method 990.10 (AOAC, 1990) [20].

**Table 2:** Primer sequences for real-time quantitative PCR.

Items ^1^	Sequence of Primers (5′-3′)	Accession Number
CA2	F: CTCCTCCGACAAGTCAGTGC R: TACGACGGCCAAACCATCAG	NM_205317.2
OC17	F: GCAATGCCTTCGTCTGCAAAG R: CTGCTGTGGGTCCGTTTATTG	XM_040659860.2
OC116	F: AAGAGCCAACATCCAAGTGGGTGAGAAT R: CAGTGACCACATGGCTCCCTTTCCT	NM_204569.1
OCX32	F: GGACAGCACTGCACTACATCAA R: GGAATTTCGTGGAGCAAGACAA	NM_204534.5
β-actin	F: GAGAAATTGTGCGTGACATCA R: CCTGAACCTCTCATTGCCA	NM_205518.2

^1.^ CA2 (carbonic anhydrase 2): the protein encoded by this gene is one of several isozymes of carbonic anhydrase, which catalyzes reversible hydration of carbon dioxide. OC17 (ovocledidin-17): OC17 is a C-type, lectin-like phosphoprotein of 17 kDa present in glycosylated (23 kDa) and non-glycosylated forms in the shell matrix. OC116 (ovocledidin-116) has been related to eggshell strength: OC-116 is a protein core of 120–200 kDa eggshell dermatan sulfate proteoglycan termed ovoglycan which is present in the compact calcified eggshell. OCX32 (Ovocalyxin-32): a matrix protein in the outer layer of the eggshell and in the cuticle, related to eggshell thickness.

**Table 3:** Effect of organic trace elements supplement on productive performance of laying hens ^1^.

Items	Groups ^2^				SEM	*p*-Value
	CON	LOT	MOT	HOT		
Egg production (%)	81.67	80.49	82.76	83.10	0.88	0.73
Average daily feed intake (g)	109.48	108.79	108.57	109.55	0.20	0.19
Daily egg mass (g)	49.39	46.48	48.19	48.59	0.56	0.48
Feed to egg ratio	2.22	2.34	2.26	2.26	0.03	0.47
Egg loss (%)	0.79	1.01	0.85	0.78	0.09	0.84

^1^ Data were the mean of six replicates (each replicate 15 hens) per treatment. ^2^ CON: normal diet supplemented with inorganic trace minerals; LOT: normal diet supplemented with peptide-chelated trace minerals at 20% regular level of inorganic trace minerals supplement; MOT: normal diet supplemented with peptide-chelated trace minerals at 30% regular level of inorganic trace minerals supplement; and HOT: normal diet supplemented with peptide-chelated trace minerals at 40% regular level of inorganic trace minerals supplement.

**Table 4:** Effect of organic trace elements supplement on serum indicators of laying hens ^1^.

Items	Groups ^2^				SEM	*p*-Value
	CON	LOT	MOT	HOT		
UA (μmol/L)	101.25	111.28	94.24	101.05	5.23	0.84
GLU (mmol/L)	13.91	14.78	14.43	12.71	0.49	0.58
T-CHO (mmol/L)	3.11	3.38	3.17	2.79	0.16	0.66
TG (mmol/L)	11.25	12.36	12.14	9.45	0.71	0.48

Abbreviation: UA, urine acid; GLU, glucose; T-CHO, total cholesterol; TG, triglyceride. ^1^ Data were the mean of six replicates per treatment. ^2^ CON: 100% commercial level inorganic trace elements; LOT: 20% commercial level organic trace elements; MOT: 30% commercial level organic trace elements; and HOT: 40% commercial level organic trace elements.

**Table 5:** Effect of organic trace elements supplement on liver function impairment of laying hens ^1^.

Items	Groups ^2^				SEM	*p*-Value
	CON	LOT	MOT	HOT		
ALB (g/L)	17.05	17.55	18.35	16.44	0.34	0.27
AST (U/L)	16.11	18.12	17.67	18.29	0.53	0.43
ALT (U/L)	5.08	5.86	5.51	5.56	0.27	0.82

Abbreviation: ALB, albumin; AST, aspartate aminotransferase; ALT, alanine aminotransferase. ^1^ Data were the mean of six replicates per treatment. ^2^ CON: 100% commercial level inorganic trace elements; LOT: 20% commercial level organic trace elements; MOT: 30% commercial level organic trace elements; and HOT: 40% commercial level organic trace elements.

**Table 6:** Effect of organic trace elements supplement on serum immunity of laying hens ^1^.

Items	Groups ^2^				SEM	*p*-Value
	CON	LOT	MOT	HOT		
IgA (mg/mL)	2.79	2.80	3.05	2.72	0.10	0.67
IgG (mg/mL)	5.49	6.29	6.72	5.65	0.26	0.33
IgM (mg/mL)	2.24	2.22	2.86	2.39	0.12	0.17

Abbreviation: IgA, Immunoglobulin A; IgG, Immunoglobulin G; IgM, Immunoglobulin M. ^1^ Data were the mean of six replicates per treatment. ^2^ CON: 100% commercial level inorganic trace elements; LOT: 20% commercial level organic trace elements; MOT: 30% commercial level organic trace elements; and HOT: 40% commercial level organic trace elements.

**Table 7:** Effect of organic trace elements supplement on serum antioxidant of laying hens ^1^.

Items ^3^	Groups ^2^				SEM	*p*-Value
	CON	LOT	MOT	HOT		
T-AOC (mmol/L)	1.28 ^ab^	1.13 ^b^	1.43 ^a^	1.15 ^b^	0.05	0.08
T-SOD (U/mL)	612.24	610.66	622.44	626.06	20.26	0.99
Cu/Zn-SOD (U/mL)	349.98	346.35	352.02	359.28	4.09	0.74
Mn-SOD (U/mL)	263.62	264.31	264.96	272.45	19.01	0.10
CAT (U/mL)	0.56 ^b^	0.87 ^a^	0.82 ^a^	0.92 ^a^	0.04	<0.01
GSH-Px (U/mL)	3726.58	3402.53	3645.57	3402.53	113.42	0.72
MDA (nmol/mL)	6.22	6.45	5.91	5.96	0.20	0.81

Abbreviation: T-AOC, total antioxidant capacity; T-SOD, total superoxide dismutase; Cu/Zn-SOD, Cu/Zn−superoxide dismutase; Mn-SOD, manganese superoxide dismutase; CAT, Catalase; GSH-Px, glutathione peroxidase; and MDA, malondialdehyde. ^1^ Data were the mean of six replicates per treatment. ^2^ CON: 100% commercial level inorganic trace elements; LOT: 20% commercial level organic trace elements; MOT: 30% commercial level organic trace elements; and HOT: 40% commercial level organic trace elements. ^3^ Mean values within the same row with different superscripts differ significantly expressed as *p* < 0.05.

**Table 8:** Effect of organic trace elements supplement on egg quality ^1^.

Items ^3^	Groups ^2^				SEM	*p*-Value
	CON	LOT	MOT	HOT		
Yolk color ^4^	6.17	6.30	6.17	5.83	0.15	0.76
Albumen height (mm)	5.59 ^b^	6.33 ^a^	6.49 ^a^	6.39 ^a^	0.13	0.04
Haugh unit ^5^	72.17 ^b^	75.33 ^ab^	77.85 ^a^	79.45 ^a^	0.92	0.03
Shell strength (N)	46.14 ^b^	44.18 ^b^	50.52 ^ab^	56.37 ^a^	1.55	0.01
Shell thickness (μm)	347.36 ^b^	347.87 ^b^	364.60 ^a^	371.57 ^a^	3.35	0.01

^1^ Data were the mean of 12 eggs per treatment. ^2^ CON: 100% commercial level inorganic trace elements; LOT: 20% commercial level organic trace elements; MOT: 30% commercial level organic trace elements; and HOT: 40% commercial level organic trace elements. ^3^ Mean values within the same row with different superscripts differ significantly expressed as *p* < 0.05. ^4^ The yolk color detection range is 1–15 grades, with higher yolk color grades indicating a higher carotenoid content in the yolk [24]. ^5^ The formula of Haugh Unit HU = 100Log(H − 1.7W^0.37^ + 7.57), H: Albumen height, and W: Egg weight.

**Table 9:** Effect of organic trace elements supplement on eggshell ultrastructure of laying hens ^1^.

Items ^3^	Groups ^2^				SEM	*p*-Value
	CON	LOT	MOT	HOT		
Mammillary layer (μm)	73.20 ^a^	68.74 ^a^	57.08 ^b^	50.86 ^b^	2.24	<0.01
Palisade layer (μm)	284.58 ^b^	287.24 ^b^	300.58 ^a^	307.25 ^a^	2.01	<0.01
Total thickness (μm)	351.78 ^b^	353.59 ^b^	362.97 ^a^	361.28 ^a^	1.42	0.01
Mammillary knob width (μm)	68.48 ^ab^	60.64 ^b^	68.15 ^ab^	73.23 ^a^	1.85	0.08
Mammillary layer (%)	20.50 ^a^	19.30 ^a^	15.94 ^b^	14.19 ^c^	0.43	<0.01
Palisade layer (%)	79.50 ^c^	80.70 ^c^	84.06 ^b^	85.81 ^a^	0.43	<0.01

^1^ Data were the mean of six replicates per treatment. ^2^ CON: 100% commercial level inorganic trace elements; LOT: 20% commercial level organic trace elements; MOT: 30% commercial level organic trace elements; and HOT: 40% commercial level organic trace elements. ^3^ Mean values within the same row with different superscripts differ significantly expressed as *p* < 0.05.

**Table 10:** The enzyme activity expression of LOX, CA, and ALP and the concentration of E2 in the serum of laying hens ^1^.

Items ^3^	Groups ^2^				SEM	*p*-Value
	CON	LOT	MOT	HOT		
LOX (IU/L)	44.42 ^b^	40.89 ^c^	45.07 ^b^	56.31 ^a^	1.11	<0.01
CA (ng/mL)	6.47 ^b^	7.40 ^ab^	8.79 ^a^	8.76 ^a^	0.35	0.03
ALP (U/L)	110.57 ^bc^	86.66 ^c^	178.69 ^ab^	196.40 ^a^	16.68	0.02
E2 (ng/L)	78.51	78.96	84.82	84.28	2.88	0.84

Abbreviation: LOX, lysyl oxidase; CA, carbonic anhydrase; ALP, alkaline phosphatase; and E2, estradiol. ^1^ Data were the mean of six replicates per treatment. ^2^ CON: 100% commercial level inorganic trace elements; LOT: 20% commercial level organic trace elements; MOT: 30% commercial level organic trace elements; and HOT: 40% commercial level organic trace elements. ^3^ Mean values within the same row with different superscripts differ significantly expressed as *p* < 0.05.

**Table 11:** Effect of organic micronutrient supplementation on the length of uterine cilia ^1^.

Items ^3^	Groups ^2^				SEM	*p*-Value
	CON	LOT	MOT	HOT		
Cilia length of uterus (μm)	5.89 ^a^	6.31 ^a^	4.85 ^b^	4.20 ^b^	0.17	<0.01

^1^ Data were the mean of three replicates per treatment. ^2^ CON: 100% commercial level inorganic trace elements; LOT: 20% commercial level organic trace elements; MOT: 30% commercial level organic trace elements; and HOT: 40% commercial level organic trace elements. ^3^ Mean values within the same row with different superscripts differ significantly and are expressed as *p* < 0.05.

**Table 12:** Effect of organic micronutrient supplementation on the content of calcium and phosphorus in eggshell and serum, respectively ^1^.

Items ^3^	Groups ^2^				SEM	*p*-Value
	CON	LOT	MOT	HOT		
Eggshell						
Calcium (%)	38.70 ^b^	38.38 ^b^	39.28 ^a^	39.59 ^a^	0.12	<0.01
Phosphorus (%)	0.39	0.40	0.40	0.40	0.003	0.41
Serum						
Calcium(mM)	5.16	4.97	4.77	4.88	0.10	0.64
Phosphorus(mM)	3.42	3.26	3.14	3.28	0.08	0.74

^1^ Data were the mean of six replicates per treatment. ^2^ CON: 100% commercial level inorganic trace elements; LOT: 20% commercial level organic trace elements; MOT: 30% commercial level organic trace elements; and HOT: 40% commercial level organic trace elements. ^3^ Mean values within the same row with different superscripts differ significantly and are expressed as *p* < 0.05.

**Table 13:** Effect of organic trace elements supplement on trace elements deposition of laying hens ^1^.

Items ^3^	Groups ^2^				SEM	*p*-Value
	CON	LOT	MOT	HOT		
Feces (mg/kg)						
Fe	636.63 ^a^	435.10 ^c^	382.68 ^c^	553.12 ^b^	23.23	<0.01
Cu	37.27 ^a^	19.12 ^b^	19.12 ^b^	24.02 ^b^	1.82	<0.01
Zn	356.80 ^a^	204.43 ^b^	209.98 ^b^	217.48 ^b^	14.52	<0.01
Mn	347.13 ^a^	135.93 ^c^	146.17 ^c^	186.8 ^b^	18.76	<0.01
Serum (mg/kg)						
Fe	42.00 ^a^	23.51 ^b^	35.98 ^b^	36.46 ^b^	1.66	<0.01
Cu	4.74 ^a^	3.24 ^b^	3.66 ^ab^	3.97 ^ab^	0.20	0.09
Zn	9.92 ^a^	7.13 ^b^	7.90 ^ab^	8.90 ^ab^	0.34	0.05
Mn	ND	ND	ND	ND		
Eggshell (mg/kg)						
Fe	5.35	4.42	4.63	5.22	0.21	0.33
Cu	0.77 ^ab^	0.97 ^a^	0.60 ^b^	0.80 ^ab^	0.05	0.11
Zn	8.66	7.44	6.24	8.83	0.46	0.26
Mn	ND	ND	ND	ND		
Yolk (mg/kg)						
Fe	148.21	136.18	141.98	150.40	3.05	0.30
Cu	5.83	5.72	5.93	5.13	0.14	0.17
Zn	131.19	131.22	125.74	130.45	1.01	0.29
Mn	1.00	0.97	1.03	1.20	0.05	0.59

^1^ Data were the mean of six replicates per treatment. ^2^ CON: 100% commercial level inorganic trace elements; LOT: 20% commercial level organic trace elements; MOT: 30% commercial level organic trace elements; and HOT: 40% commercial level organic trace elements. ND: not detected, the concentration of Mn was below the threshold of the instrument. ^3^ Mean values within the same row with different superscripts differ significantly and are expressed as *p* < 0.05.

## Data Availability

The 16S rDNA gene sequence data have been deposited tin the NCBI BioProject database https://www.ncbi.nlm.nih.gov/bioproject/PRJNA1074713 and https://www.ncbi.nlm.nih.gov/bioproject/PRJNA107472 (accessed on 8 February 2024).
